# Dengue in the Context of the Integrated Management of Childhood Illness

**DOI:** 10.1371/journal.pntd.0004838

**Published:** 2016-08-25

**Authors:** Jacqueline Deen, Martin W. Weber, Thomas Jaenisch

**Affiliations:** 1 Department of Pediatrics, Vicente Sotto Memorial Medical Center, Cebu City, Philippines; 2 WHO Regional Office for Europe, UN City, Copenhagen, Denmark; 3 Section Clinical Tropical Medicine, Department for Infectious Diseases, Heidelberg University Hospital, Heidelberg, Germany; Pediatric Dengue Vaccine Initiative, UNITED STATES

## Background

The WHO/UNICEF Integrated Management of Childhood Illness (IMCI) is widely used for the management of common childhood conditions, including cough and difficulty breathing, diarrhoea and dehydration, and fever [1]. Dengue fever is not included in the generic IMCI, but in view of its public health importance, various empiric dengue fever algorithms have been incorporated into national IMCI adaptations [2]. These algorithms vary in terms of assessment criteria, dengue illness categories, and management and have, with few exceptions [3], not been validated. Should a standard, evidence-based and validated dengue fever algorithm be developed for integration into IMCI in dengue-endemic areas?

## A Potential Dengue Fever IMCI Algorithm

How useful would such an algorithm be? Dengue fever is a major concern for parents with febrile children in several tropical Asian countries. About 32% of reported dengue fever cases in Cambodia in 2010, 25% in Lao PDR from 2000 to 2006, 19% in the Philippines from 1998 to 2005, and 12% in Sri Lanka from 1996 to 2005 were in children less than five years of age [4]. It would be important for health workers to consider this diagnosis when sick children are brought for care in areas where childhood dengue is common. Because IMCI primarily targets children under five years of age, inclusion of dengue fever in IMCI would only cover a fraction of cases in the population. However, fever is one of the most frequent presenting symptoms in first-line centres, which translates into high numbers of children who need to be assessed, classified, and treated. The inclusion of a dengue fever algorithm into IMCI in areas where childhood dengue is common may facilitate its differentiation from other febrile diseases and decrease the chances of missing this important diagnosis. Standardised assessment of dengue fever may also improve surveillance and reporting. Finally, inclusion of dengue fever in IMCI may increase health worker adherence to IMCI strategies in areas with high dengue transmission.

One important component in the development of a standard IMCI dengue fever algorithm is the warning signs for severe disease. A study that followed nearly 2,000 dengue fever patients aged six months and older in Asia and Latin America assessed warning signs in the 5% of patients who progressed to severe disease [5]. Although the study was primarily designed to provide evidence for the WHO 2009 dengue fever guidelines [6], the results with regard to warning signs may be useful for the development of a standard IMCI dengue fever algorithm. However, the positive and negative predictive values of many warning signs remain undetermined. Patients presenting to hospitals with severe dengue may have missed being referred earlier by primary health care workers or may not have presented any warning signs. Large prospective studies are currently underway to empirically validate warning signs for severe dengue [7,8].

A potential dengue fever IMCI algorithm for use by first-line health care workers will need to include clear instructions on immediate referral or daily follow-up based on the presence of warning signs. For patients who will be sent home, messages for the caretakers on supportive care, follow-up, and warning signs to look out for should be developed. Key messages on the potential of oral rehydration need to be incorporated. In a study in Nicaragua, intake of fluids (most commonly water, fruit juices, and oral rehydration solution) during the 24 hours before being seen by a clinician was statistically associated with a decreased risk for hospitalization of dengue fever patients [9]. This algorithm should not duplicate existing guidelines on administration of IV fluids in hospital settings [6,10], but focus on the initial management in primary health centres.

Information about the timing of possible complications (which often evolve around the time of defervescence [6,10]) should be included in the algorithm, as well as expected changes of key laboratory results—for example, white blood cell count, haematocrit, and platelet count. However, even simple laboratory tests may not be available in some first-line treatment centres. It will be important to clearly indicate when to refer patients for a complete blood count (CBC) and dengue laboratory confirmation. Similar to the IMCI malaria algorithm [11], it may be feasible to incorporate dengue rapid diagnostic tests in the future as their price drops and accuracy improves.

Once the dengue fever IMCI algorithm is tested and validated, the next important task would be rollout, harmonization, and implementation across dengue-endemic countries. Widespread implementation of an evidenced-based dengue algorithm will be challenging and requires addressing perceptions and expectations of health workers and patients alike. For this process to be successful, skilful communication tools have to be applied. The algorithm to be implemented needs to be simple and highly efficient in order convince health care workers in resource-poor settings of its usefulness.

The peak age group affected by dengue varies by country, but dengue can occur in all age groups. National experts may need to be consulted to further qualify the algorithm based on the local disease epidemiology. With adequate support, research could be carried out to explore an extension of the age range of IMCI to older children. A standard dengue fever algorithm could be incorporated in the Integrated Management of Adult and Adolescent Illness (IMAI) and used in parallel with IMCI. Analogous to IMCI, IMAI consists of a series of simplified, syndromic protocols to diagnose and manage common adult illnesses but has received little attention [12].

## Conclusion

We believe that incorporation of a dengue fever algorithm into IMCI would be beneficial. Aside from contending with age limitations currently inherent in IMCI, there are several challenges in terms of developing appropriate instructions for first-line health workers and messages for the mother or caretaker. Widespread dissemination of good outpatient dengue guidelines may be one step towards improving primary care delivery in dengue hyper-endemic settings.
